# Correction: Mothers’ experiences of using Facebook groups for local breastfeeding support: Results of an online survey exploring midwife moderation

**DOI:** 10.1371/journal.pdig.0000160

**Published:** 2022-11-23

**Authors:** The PLOS Digital Health Staff

## Notice of republication

This article was republished on November 11, 2022 to correct a typographical error in the title. The publisher apologizes for the error. Please download this article again to view the correct version.
