# Correction: Mothers’ experiences of using Facebook groups for local breastfeeding support: Results of an online survey exploring midwife moderation

**DOI:** 10.1371/journal.pdig.0000212

**Published:** 2023-02-24

**Authors:** Holly Morse, Amy Brown

In Table 1, the number and percentage values in the Indicator “Age” are incorrect. Please see the correct Table 1 below.

**Table 1:** Sample distribution by demographic factors.

Indicator	Group	N	%
Age	≤ 20	9	0.4
	21–25	127	6.3
	26–30	546	27.2
	31–35	826	41.1
	36–40	418	20.8
	41 ≥	62	3.1
	Missing	23	1.1
Education	No formal	11	0.6
	GCSE or equivalent	117	5.8
	A-Level or equivalent	307	15.2
	Degree or equivalent	883	43.9
	Postgraduate or equivalent	692	34.4
Ethnicity	Asian or Asian British (Chinese, Indian, Bangladeshi, Pakistani, Other)	47	2.34
	Black or Black British	5	0.25
	Gypsy/Traveller	1	0.05
	Irish	35	1.74
	Mixed or multiple	41	2.04
	Other	12	0.60
	White/White British	1872	93.0
Marital Status	Married/civil partnership	1451	72.2
	Divorced	10	0.50
	Cohabiting	474	23.6
	Single	73	3.6
	Widowed	2	0.10
			
Employment	Full time	819	40.8
	Part time	828	41.2
	Not working	361	18.0
			
